# Giant left main coronary artery aneurysm with fistulous drainage to the right atrium: a rare case report and surgical management approach

**DOI:** 10.1186/s13019-026-03965-6

**Published:** 2026-04-04

**Authors:** Nuha Riyad, Jihad Jaara, Mahmoud Abdelrazzaq Abu Mayaleh, Ahmad Darwazah, Ahmed Motawe, Mahmoud Mansour, Omar Al-Haj, Abdallah Abu Zant, Basem Bail, Mohammad N. Sbaih, Osama Ewidat

**Affiliations:** 1 Department of Medicine, Faculty of Medicine and Health Sciences, An- Najah National University, Nablus, Palestine; 2 Nablus Specialty Hospital, Nablus, Palestine; 3 College of Medicine, Hebron University, Hebron, Palestine; 4 Palestine Medical Complex, Ramallah, Palestine; 5 British Medical Center, Nablus, Palestine; 6 Department of Surgery, Faculty of Medicine and Health Sciences, An-Najah National University, Nablus, P600 Palestine

**Keywords:** Left main coronary artery, Coronary artery aneurysm, Coronary artery fistula, Right atrium, Surgical ligation, Atrial fibrillation, Sick sinus syndrome, Case report

## Abstract

Coronary artery aneurysm (CAA) is defined as a focal dilation that exceeds 1.5 times the diameter of adjacent normal coronary segments. Involvement of the left main coronary artery (LMCA), particularly when associated with a fistulous communication to the right atrium (RA), is exceedingly rare. We report a case of a 55-year-old male with a giant LMCA aneurysm and a coronary artery fistula (CAF) draining into the RA, diagnosed via coronary angiography and contrast-enhanced CT angiography. The patient underwent surgical closure of the LMCA-RA fistula. Following discharge, he developed worsening respiratory symptoms, and electrocardiography revealed atrial fibrillation, which later progressed to symptomatic bradycardia. Sick sinus syndrome was diagnosed, and a permanent pacemaker was successfully implanted. This case underscores the critical importance of early diagnosis and definitive surgical repair of LMCA aneurysms with fistulous connections, as well as the need for vigilant monitoring of potential postoperative complications such as arrhythmias, given the hemodynamic implications of left-to-right shunting and subsequent atrial stretch.

## Introduction

Coronary artery aneurysm (CAA) is defined as a localized dilation of a coronary artery segment measuring more than 1.5 times the diameter of adjacent normal segments [1]. Its mean angiographic incidence is estimated at 1.65%, with reported ranges between 0.3% and 5.3% [2]. Among these, left main coronary artery (LMCA) aneurysms are particularly uncommon, with a reported incidence of approximately 0.1% in large angiographic series [3]. An even rarer presentation is the coexistence of a CAA with a coronary artery fistula (CAF) an anomalous communication between a coronary artery and a cardiac chamber or vascular structure. CAFs are found in only 0.002% of the general population and 0.2% of patients undergoing coronary angiography. LMCA involvement occurs in approximately 0.7% of all CAF cases, making the combination of LMCA aneurysm and fistula exceptionally rare [4].

The etiology of CAAs is heterogeneous and may include congenital defects, atherosclerosis, inflammatory vasculitis (e.g., Kawasaki disease), infections, trauma, or iatrogenic injury [5]. LMCA-RA fistulas are predominantly congenital in origin, arising from a failure of embryonic coronary artery development to regress, though acquired causes are also recognized [6]. The continuous high-pressure shunting through these anomalous connections leads to progressive dilation of the feeding coronary artery and subsequent aneurysm formation due to chronic hemodynamic stress on the vessel wall [7].

When a CAF and aneurysm coexist, the risk of severe complications—including myocardial ischemia, rupture, or thrombosis increases significantly, and such cases often require prompt surgical correction [8]. The co-occurrence of a coronary artery aneurysm with a left-to-right coronary shunt significantly elevates the risk of a broad spectrum of serious complications. These include myocardial ischemia due to the “coronary steal” phenomenon, progressive heart failure from chronic volume overload on the right heart, infective endocarditis, and potentially life-threatening events such as aneurysm rupture or thrombosis [9, 10]. Recognizing this wide range of potential adverse outcomes underscores the critical importance of early diagnosis and timely intervention for these combined anomalies.

## Case presentation

**Fig. 1:**
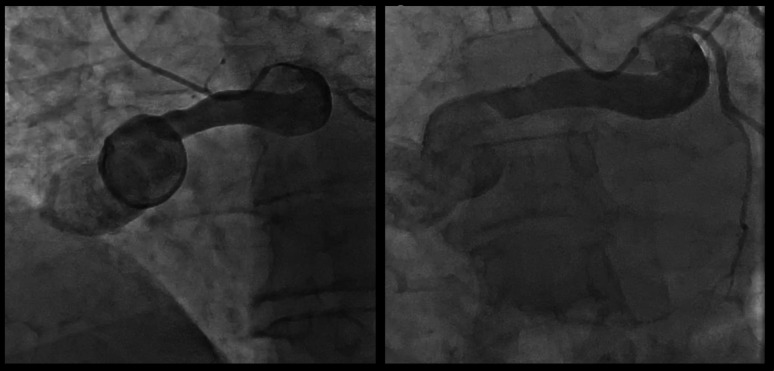
Selective left coronary angiography in two orthogonal projections demonstrating a giant saccular aneurysm of the left main coronary artery (LMCA) with continuity into the proximal left circumflex artery (LCX). There is opacification of a large abnormal vascular channel extending from the aneurysmal sac toward the right atrium, consistent with a left-to-right coronary artery fistula. The contrast-filled sac and uninterrupted dye column confirm patency of the fistulous tract at the time of angiography

A 55-year-old male with a history of hypertension presented to our hospital for elective coronary angiography as he complained of exertional dyspnea and chest discomfort. Coronary angiography was performed and revealed a huge left main coronary artery aneurysm with a suspicious fistula tract, the mid-left anterior descending artery demonstrated mild to moderate stenosis, while the right coronary artery was angiographically non-visualized [Fig. 1].

**Fig. 2:**
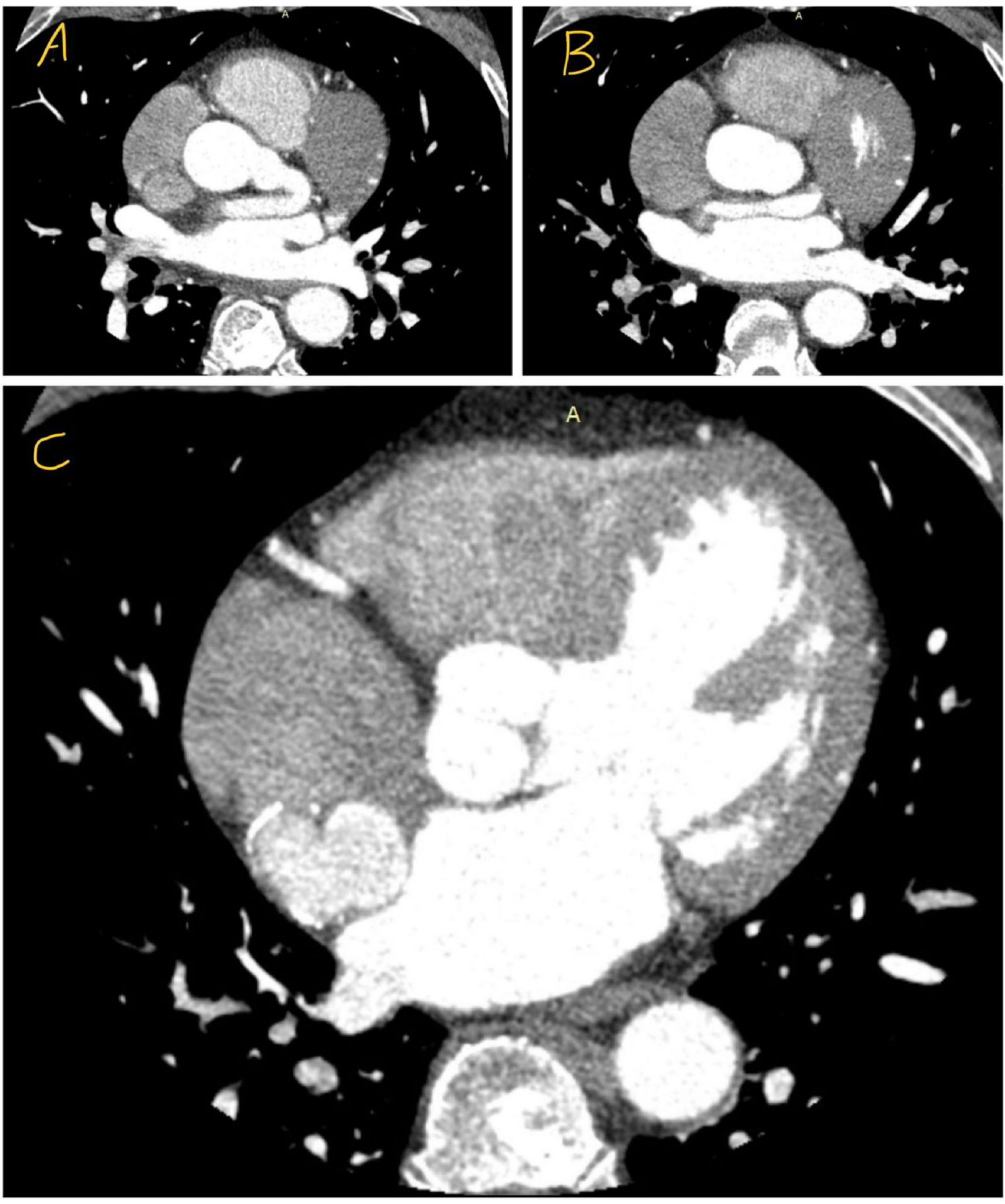
CT coronary angiography demonstrating a giant left main coronary artery aneurysm with fistulous drainage to the right atrium

**Fig. 3:**
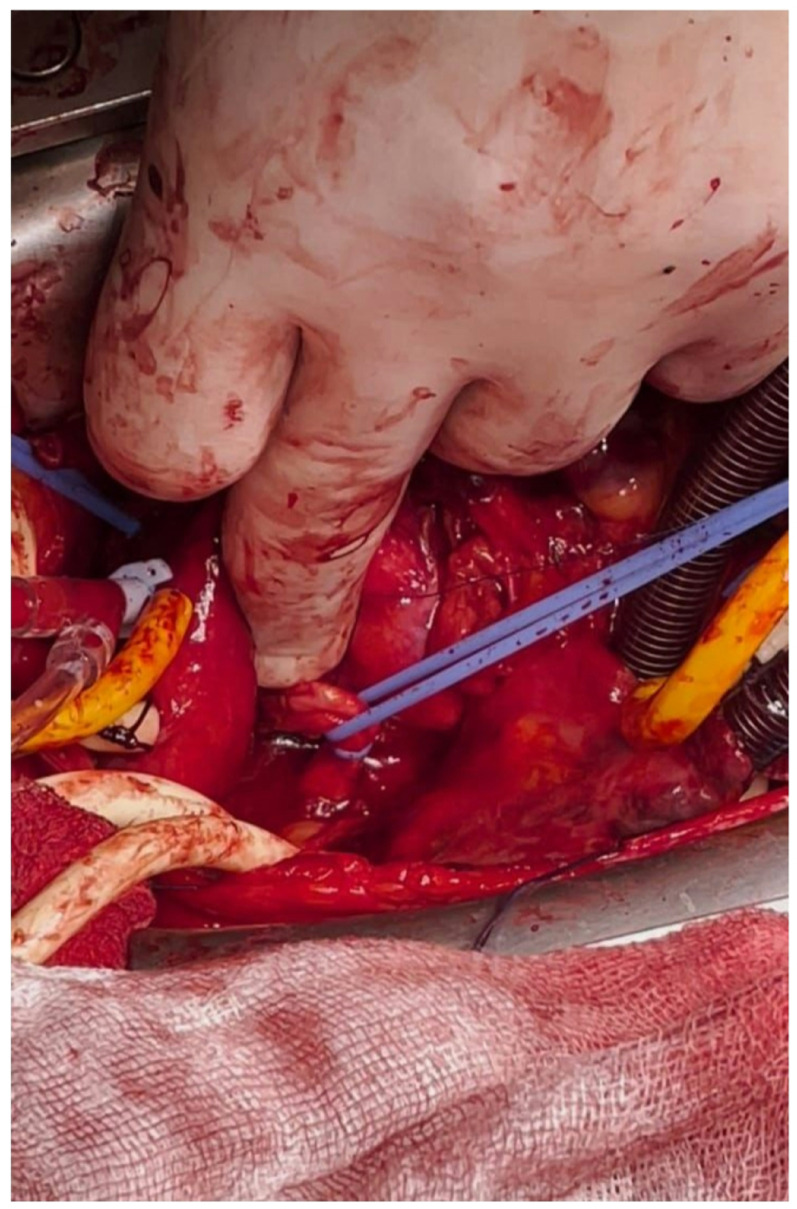
Intraoperative photograph showing the aneurysmal segment of the left main coronary artery (LMCA) and the fistulous connection to the right atrium, encircled with blue tourniquet for proximal control. A gloved finger indicates the site of the fistulous origin, prior to closure. The procedure was performed on-pump with a beating heart. This image demonstrates the complex anatomy and meticulous surgical dissection required to preserve LMCA patency while excluding the fistula

**Fig. 4:**
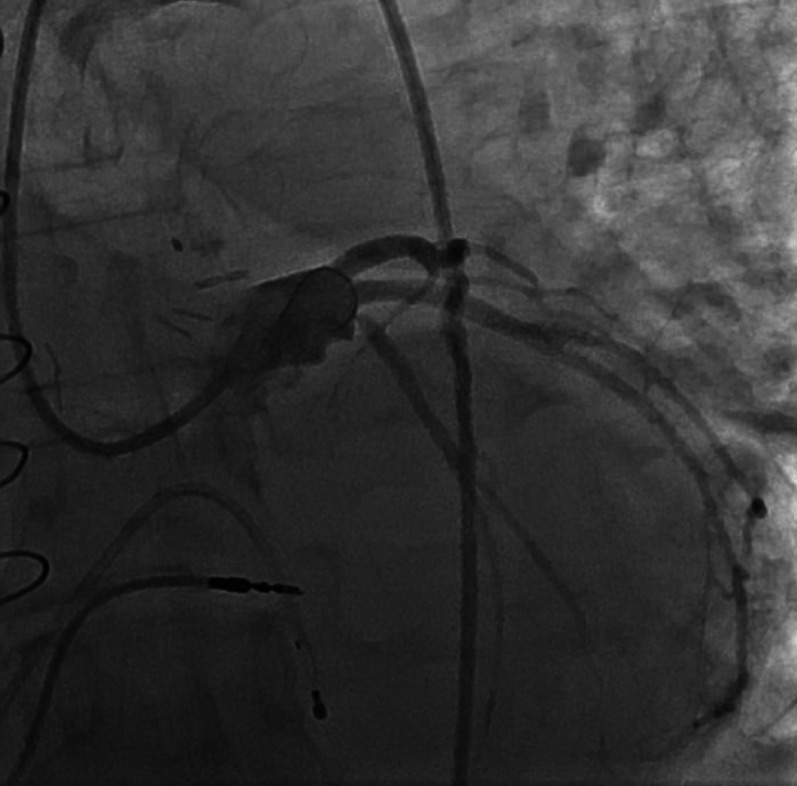
Coronary angiography demonstrating complete opacification of the original left main coronary artery (LMCA) to right atrium fistula tract and revealing a normal-caliber LMCA. Note the newly unmasked severe ostial stenosis of the left circumflex artery (LCX), which had been previously obscured by the aneurysmal segment of the LMCA

A contrast-enhanced CT coronary angiography was performed for further detailed anatomical delineation of the coronary anatomy, aneurysmal segment, and fistulous tract. The CT study demonstrated a giant aneurysmal dilatation of the LMCA extending into the left circumflex artery, terminating in a saccular structure with mural calcification. Additionally, a fistulous communication between the distal aneurysmal segment and the right atrium was identified. Based on contrast-enhanced CT coronary angiography, the left main coronary artery demonstrated marked aneurysmal dilatation with an estimated maximal diameter in the range of approximately 30–35 mm. Precise delineation of the true aneurysmal margins was partially limited by high-flow contrast washout into the large fistulous tract draining into the right atrium, which resulted in preferential opacification of the fistula relative to the aneurysmal sac. Therefore, the reported dimensions should be regarded as approximate rather than exact measurements, representing a limitation inherent to the hemodynamic characteristics of this lesion. The remaining coronary anatomy was otherwise unremarkable, with a right-dominant circulation and no evidence of calcified atherosclerosis within the major vessels. These findings were reviewed in a multidisciplinary setting, and the patient was counseled regarding the need for surgical correction [Fig. 2].

Preoperatively, the patient was in sinus rhythm with no documented baseline conduction abnormalities or history of bradyarrhythmia. The patient subsequently underwent elective surgical repair under cardiopulmonary bypass on a beating heart. A retro-aortic approach was used to expose the aneurysmal left main coronary artery and the fistulous tract. Intraoperatively, the fistula was identified originating from the distal segment of the aneurysmal left main coronary artery and draining directly into the right atrium. The fistulous tract was carefully dissected, and proximal control was achieved to preserve uninterrupted left main coronary artery flow. The fistula was ligated proximally at its origin using a 4 − 0 polypropylene suture, followed by closure of the distal opening at the right atrial side. Intraoperative assessment confirmed complete exclusion of the fistulous connection with preservation of left main coronary artery patency and stable hemodynamic and electrical stability throughout the procedure [Fig. 3].

The patient had an initially uneventful postoperative recovery and was discharged from the hospital in stable condition on the 3rd post-operative day. However, he re-presented to Emergency room with palpitations and hypoxia. The diagnosis of Atrial fibrillation and Pneumonia was considered, he was maintained on medical treatment for both conditions. Subsequently, the patient developed sinus node dysfunction with symptomatic bradycardia, manifesting as dizziness and fatigue. A permanent pacemaker was implanted via transvenous approach without complications, coronary angiography was performed which showed complete opacification of the original LMCA fistula tract, normal diameter of the LM coronary artery and dramatically significant ostial stenosis of the Circumflex artery which was hidden by the original aneurysmal dilatation of the left main coronary artery **[**Fig. 4**]**. The patient expressed relief after the definitive surgical treatment and pacemaker implantation. He appreciated the close follow-up and multidisciplinary care that helped manage his complications and restore his quality of life.

The Table 1 is a illustrates the chronological sequence of the patient’s clinical course, from initial presentation to post-surgical follow-up.

**Table 1:** Illustrates the chronological sequence of the patient’s clinical course, from initial presentation to post-surgical follow-up

Date / timeline	Clinical event
Baseline	55-year-old male with hypertension presented with exertional dyspnea and chest discomfort
Day 0	Underwent elective coronary angiography → Revealed giant LMCA aneurysm with suspected fistula
Day 2	Cardiac CT angiography confirmed LMCA aneurysm and fistulous drainage into the right atrium
Day 4	Multidisciplinary heart team discussion; surgical correction was planned
Day 7	Surgical ligation of the LMCA-RA fistula performed on-pump with beating heart
Post-op day 3	Discharged home in stable condition
Post-op week 2	Re-presented to ER with palpitations and hypoxia → Diagnosed with atrial fibrillation and pneumonia
Post-op week 3	Developed symptomatic bradycardia due to sick sinus syndrome → Permanent pacemaker implanted
Follow-up	Post-pacemaker angiography showed no residual flow in the fistula; patient discharged in stable condition

## Discussion

Coronary artery fistulas (CAFs) in general, and specifically those originating from the left main coronary artery (LMCA) and draining into the right atrium (LMCA-RA), are exceedingly rare and complex anomalies [1]. These malformations, whether congenital or acquired, bypass the myocardial capillary bed. When associated with aneurysmal dilation, they may lead to severe complications such as arrhythmias, myocardial infarction, and sudden death [8].

Coronary angiography remains the gold standard for the diagnosis of CAFs. Advanced imaging modalities such as cardiac computed tomography (CT) angiography and echocardiography are often necessary when complex draining pathways and aneurysmal dilatations are present [11].

The inherent left-to-right shunting in coronary artery fistulas, particularly large ones draining into the right atrium, leads to significant volume overload on the right heart, increasing pulmonary blood flow and potentially causing right ventricular dilation [11]. Quantitative assessment of the left-to-right shunt using the pulmonary-to-systemic flow ratio (Qp/Qs) was not performed in this case, which represents a limitation of this case report. Shunt significance was instead inferred from clinical presentation, imaging findings, and intraoperative assessment. The patient’s presenting symptoms, including exertional dyspnea and chest discomfort, were likely multifactorial in origin. Myocardial ischemia secondary to the coronary steal phenomenon may have contributed, as blood was preferentially diverted through the low-resistance fistulous pathway rather than perfusing the myocardial capillary bed. In parallel, chronic volume overload resulting from a high-flow left-to-right shunt into the right atrium may have led to heart failure physiology, further explaining the patient’s clinical presentation. This continuous shunting can also result in a “coronary steal” phenomenon, where blood preferentially flows into the low-pressure right atrium rather than adequately perfusing the myocardial capillary bed, potentially leading to myocardial ischemia [12, 13]. This detailed understanding of the hemodynamic alterations provides a clearer physiological basis for the patient’s presenting symptoms, such as dyspnea and chest discomfort, and underscores the rationale for surgical intervention.

Surgical ligation remains the standard treatment for large, hemodynamically significant coronary artery fistulas, particularly when aneurysmal dilation is present. Although percutaneous transcatheter closure is a recognized option for coronary artery fistulas, it was not pursued in this case due to the giant size of the LMCA aneurysm, the complex fistulous tract morphology, and the risk of incomplete closure or residual shunting. Therefore, surgical ligation was selected to ensure definitive repair with direct visualization and anatomical control.

The patient’s case was complicated by atrial fibrillation, bradycardia, and sick sinus syndrome, consistent with literature on atrial stretch due to left-to-right shunts [14]. Although ligation of the fistulous connection reduces atrial volume overload, chronic atrial stretch may result in irreversible structural and electrophysiological remodeling of the atrial myocardium and sinoatrial node. This mechanism may explain the persistence or clinical unmasking of sinus node dysfunction following fistula closure, ultimately leading to the development of sick sinus syndrome in this patient. The chronic right heart volume overload causes sustained atrial stretch, leading to electrophysiological remodeling in the atrial tissue and sinoatrial node. This remodeling predisposes to atrial fibrillation and progressive sinus node dysfunction, manifesting as tachy-brady syndrome, which required a permanent pacemaker. This mechanistic explanation supports the patient’s post-operative arrhythmic complications.

This case contributes to the expansion of the limited literature on LMCA-to-RA CAFs. Beyond the rarity of an LMCA-to-right atrium coronary artery fistula with aneurysmal dilatation, this case is notable for its complex postoperative course. Unlike previously reported cases that primarily focused on anatomical correction and hemodynamic outcomes, the present case illustrates the sequential development of atrial fibrillation, progressive sinus node dysfunction, and symptomatic bradycardia requiring permanent pacemaker implantation. Additionally, this case provides a clinical insight into how severe aneurysmal distortion of the left main coronary artery can mask significant ostial coronary stenosis, which only became evident following surgical decompression and normalization of coronary anatomy. Comparison with previously reported cases allows contextualization of the present findings within the existing literature and highlights relevant clinical similarities and differences.

A case of a 43-year-old male presenting with a giant aneurysmal LMCA-RA fistula measuring 4.3 cm×3.5 cm, causing congestive heart failure symptoms and a high cardiac output state with likelihood of coronary steal phenomenon, was successfully managed surgically [15]. Another report detailed the surgical management of an LMCA-RA fistula with a saccular aneurysm measuring 34 × 37 mm, emphasizing the approach of fistula division and obliteration, leading to an uneventful recovery [16]. These examples highlight similarities in presentation and management, reinforcing the validity of the surgical approach for such large and complex anomalies. Presenting these comparisons in a structured format, such as Table 2, can further enhance clarity and utility for the reader.

**Table 2:** Comparative Cases of LMCA-RA Fistula

Feature	Current case (patient in report)	Comparative case 1	Comparative case 2
Patient age/sex	55-year-old male	43-year-old African American male	Not specified
Fistula origin	Left Main Coronary Artery (LMCA)	Left Main Coronary Artery (LMCA)	Left Main Coronary Artery (LMCA)
Fistula termination	Right Atrium (RA)	Right Atrium (RA)	Right Atrium (RA)
Aneurysm size	Giant LMCA aneurysm	Giant aneurysmal fistula (4.3 cm×3.5 cm)	Saccular aneurysm (34 mm×37 mm)
Key symptoms/presentation	Exertional dyspnea, chest discomfort, palpitations, hypoxia	Congestive heart failure symptoms, high cardiac output	Angina, marked left-to-right shunt
Management approach	Surgical closure of LMCA-RA fistula	Surgical ligation of fistulous tract	Surgical division and obliteration of fistula
Key complications	Atrial fibrillation, sick sinus syndrome, symptomatic bradycardia, pneumonia, need for permanent pacemaker	Coronary steal phenomenon	Risk of saccular aneurysm rupture, potential for device migration (if percutaneous)
Outcome/follow-up	Discharged stable post-pacemaker implantation	Uneventful post-operative course, thrombosed fistula at 3 weeks	Asymptomatic and free of adverse events at 6 months

Prompt diagnosis and early surgical intervention are indeed paramount for large or symptomatic LMCA-RA fistulas, even in asymptomatic patients, to prevent severe complications and address the underlying hemodynamic burden [17]. Furthermore, vigilant, long-term post-operative monitoring is essential to detect and manage potential delayed complications. These complications can include residual shunts, which are not uncommon even after surgical repair (reported in up to 17% of cases), the development of new myocardial ischemia (as seen in some long-term follow-up studies) [18], or the persistence or recurrence of arrhythmias. While universal guidelines for long-term monitoring are not explicitly defined [19], individualized and diligent follow-up is crucial for ensuring sustained positive outcomes and addressing any evolving issues.

## Conclusion

This case study highlights the diagnostic and therapeutic urgency in the management of LMCA-RA fistulae. Surgical intervention continues to be the keystone in symptomatic or large fistulae, offering definitive repair. Furthermore, this case underscores the critical importance of vigilant long-term monitoring for potential postoperative complications, particularly cardiac arrhythmias, which can arise from chronic hemodynamic stress on the atrial chambers.

## Data Availability

No datasets were generated or analysed during the current study.

## Abbreviations

AF: Atrial Fibrillation
CAF: Coronary Artery Fistula
CAA: Coronary Artery Aneurysm
CT: Computed Tomography
LMCA: Left Main Coronary Artery
LCX: Left Circumflex Artery
RA: Right Atrium
SSS: Sick Sinus Syndrome
